# Targeted Biologic Therapies for Hidradenitis Suppurativa

**DOI:** 10.3390/ijms26188887

**Published:** 2025-09-12

**Authors:** Isabella J. Tan, Helen N. Nguyen, Sydney M. Wolfe, Priya Agarwal, Bernard A. Cohen

**Affiliations:** 1 Robert Wood Johnson Medical School, Rutgers, The State University of New Jersey, Piscataway, NJ 08901, USA; 2 Rutgers New Jersey Medical School, Rutgers, The State University of New Jersey, Newark, NJ 07103, USA; 3 Department of Dermatology, The Johns Hopkins Hospital, Baltimore, MD 21287, USA

**Keywords:** chronic inflammatory disorders of the apocrine gland, hidradenitis suppurativa, targeted therapy

## Abstract

Chronic inflammatory disorders of the apocrine gland (CIDAP), such as hidradenitis suppurativa (HS), are characterized by painful, recurrent lesions in apocrine gland-rich areas. First-line treatments—including retinoids and antibiotics—often fail to prevent recurrence and biofilm formation, necessitating the use of targeted biologic therapies. This review evaluated U.S.-based randomized controlled trials and cohort studies published between 2014 and 2024 on the efficacy of such therapies in adult HS patients. A total of 13 studies met inclusion criteria. Agents targeting interleukins (IL-17A, IL-17F, IL-23, IL-1α, IL-36) and TNF-α were assessed, with outcomes including HiSCR, Sartorius scores, DLQI, and patient-reported measures. IL-17 inhibitors (secukinumab, bimekizumab) and IL-1 inhibitors (bermekimab, anakinra) demonstrated promising reductions in inflammatory burden and improved quality of life. TNF-α inhibitors, particularly adalimumab and infliximab, consistently achieved HiSCR and HSS improvements. Guselkumab (IL-23) showed limited efficacy in HiSCR but modest pain relief. Safety profiles were generally acceptable across agents, with few serious adverse events. Limitations across studies included small sample sizes, lack of control arms, and short follow-up durations. These findings underscore the therapeutic potential of biologic agents in managing HS. A greater emphasis on biomarker-guided treatment selection and interdisciplinary collaboration is warranted to optimize long-term outcomes for patients.

## 1. Introduction

Chronic inflammatory disorders of the apocrine gland (CIDAP) encompass conditions such as hidradenitis suppurativa (HS). HS lesions begin around terminal hair follicle units in intertriginous areas, where the hair follicle, sebaceous gland, and apocrine gland converge. Initial changes include peri-follicular immune cell infiltration, epithelial thickening, and hyperkeratinization, which leads to follicular occlusion, cystic expansion, and secondary inflammation [1]. Its prevalence approaches 4% but is likely underestimated due to underdiagnosis. It predominantly affects women and is linked to obesity, metabolic syndrome, and smoking [1].

First-line treatments for HS include topical or oral antibiotics. Relapse and recurrence of lesions are common and can cause ongoing pain and inflammation, putting patients at risk for the development of antibiotic resistance and a chronic layer of bacteria and microbes resulting in biofilm formation [2]. Targeted treatments, including immunotherapy, which utilize biological antibodies to counteract inflammation can limit relapse and disease progression. While monoclonal antibodies and small-molecule drugs are the most common therapeutic classes, hormonal therapies, signal transduction inhibitors, gene expression modulators, apoptosis inducers, angiogenesis inhibitors, immunotherapies, and toxin delivery molecules offer alternative treatment pathways [3,4,5].

Although HS and severe acne share inflammatory pathways, HS presents distinct clinical and therapeutic challenges. The predominance of repurposed or reformulated agents underscores the urgent need for HS-specific therapies. This review synthesizes published evidence on biologic therapies for HS, encompassing both approved and investigational agents targeting extracellular cytokines or receptors.

## 2. Methods

### 2.1. Study Design and Eligibility Criteria

Studies were included if they evaluated targeted therapies related to HS and were published within the last 10 years (2014–2024). The inclusion criteria focused on the primary literature involving adult patients (>18 years old), conducted in vivo in human subjects, and restricted to studies conducted in the United States. This restriction was applied to maintain consistency in healthcare systems, population demographics, and regulatory frameworks; however, we acknowledge that it may limit the generalizability of our findings to international populations. Only randomized controlled trials (RCTs) and cohort studies assessing efficacy were included if they reported primary or secondary endpoints using commonly accepted measures for assessing improvement and severity, such as the Investigator’s Global Assessment (IGA), Patient’s Global Assessment (PGA), Dermatology Life Quality Index (DLQI), Hidradenitis Suppurativa Quality of Life (HiSQOL), Sartorius score, Hidradenitis Suppurativa Clinical Response (HiSCR), and Skindex-29.

Exclusion criteria encompassed studies evaluating non-CIDAP conditions (e.g., severe acne), secondary sources (e.g., narrative reviews, systematic reviews, or meta-analyses), case reports, case series, retrospective reviews, non-English articles, and studies not including human subjects (e.g., skin biopsies). Non-peer-reviewed sources were also excluded. The review focused on studies of at least fair quality, as determined by the Cochrane Risk of Bias checklist tool.

### 2.2. Literature Search

A comprehensive literature search was conducted in PubMed, Embase, and ClinicalTrials.gov databases from 2014 to 2024. The search strategy employed a combination of relevant keywords and title or abstract terms related to HS. The search string was tailored to the specific requirements of each database (Table S1 in the Supplementary Materials). Additionally, reference lists of included studies and relevant reviews were manually searched to identify any additional relevant articles.

### 2.3. Study Selection

Two independent reviewers (I.J.T. and H.N.N.) screened titles and abstracts to identify potentially eligible studies. Full-text articles were then assessed for eligibility based on the predefined inclusion and exclusion criteria. Any discrepancies between reviewers were resolved through discussion or by consulting a third reviewer.

### 2.4. Data Extraction

Data extraction was performed using a standardized template to capture relevant study characteristics, patient demographics, intervention details, outcomes assessed, and adverse events. Quality assessment of included studies was conducted using appropriate tools, including the Oxford Center for Evidence-Based Medicine Levels of Evidence.

### 2.5. Data Synthesis and Analysis

Data from included studies were synthesized narratively, providing a comprehensive overview of the evidence on targeted therapies for CIDAP. Results were stratified based on drug classes currently approved versus investigational mechanisms not yet approved.

## 3. Results

**Figure 1:**
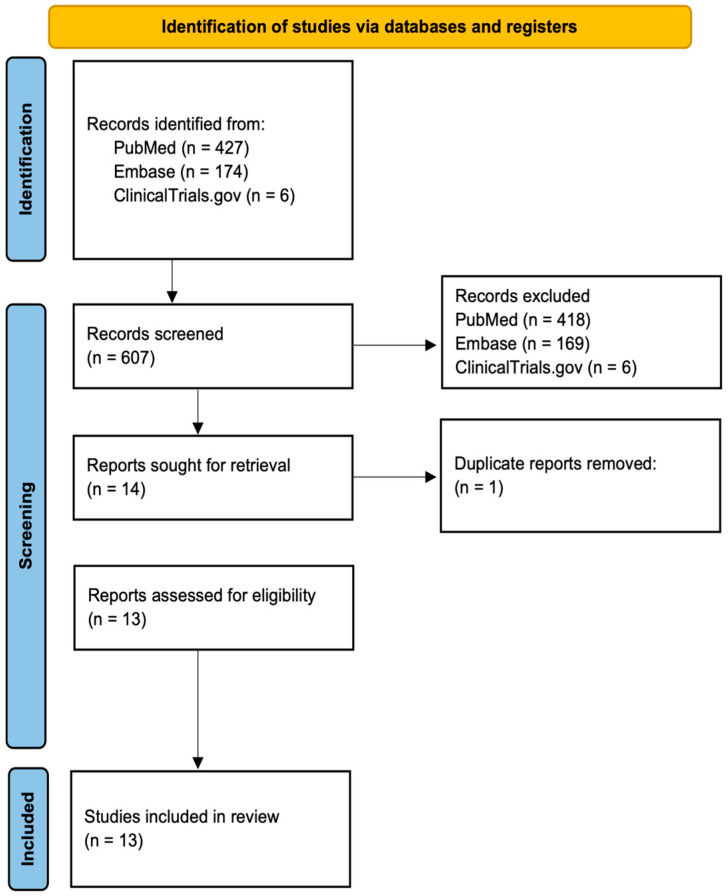
PRISMA flow diagram depicting study selection.

A comprehensive literature search yielded a total of 607 records, comprising 427 from PubMed, 174 from Embase, and 6 from ClinicalTrials.gov. After removing duplicates and screening titles and abstracts, 13 studies met the inclusion criteria and were included in this review. The study selection process is detailed in the PRISMA flow diagram (Figure 1).

The included studies evaluated the efficacy and safety of various targeted biologic therapies for the treatment of HS. The investigated therapies encompassed a range of mechanisms, including inhibition of interleukins (IL-17A, IL-17F, IL-23, IL-1α, IL-36), and tumor necrosis factor-alpha (TNF-α). Small-molecule kinase inhibitors, including JAK–STAT pathway inhibitors, were excluded, as this review focused on biologic agents targeting extracellular cytokines or receptors. Study design varied from randomized controlled trials (RCTs) to open-label and prospective cohort studies, with sample sizes ranging from 6 to over 1000 participants (Table 1).

## 4. Efficacy Outcomes

### 4.1. Secukinumab

Two open-label studies evaluated secukinumab in moderate-to-severe HS [1,2]. HS has been associated with imbalances in T-helper 17 (Th17) cells [1]. Secukinumab, a human monoclonal IgG1κ antibody, selectively binds IL-17A, reducing circulating levels [1].

Casseres et al. [1] enrolled 20 Hurley stage II–III patients, including 6 with prior anti-TNFα exposure, randomized to dosing every two or four weeks following a 5-week loading phase. HiSCR was achieved in 70% at week 24, with early responses in most patients. Sartorius and DLQI scores improved, though DLQI gains at week 24 were not statistically significant. The absence of a control group and small sample size limit internal validity, and the use of two dosing schedules complicates interpretation of dose–response relationships.

Prussick et al. [2] tested the psoriasis-approved regimen (300 mg subcutaneously (SC) weekly × 5, then every 4 weeks) in nine patients. HiSCR was achieved by 67% at week 16, with improvements in pain and lesion counts. However, without a comparator and given the small sample, conclusions on efficacy and optimal dosing remain tentative; higher dosing frequency may be warranted given HS’s higher inflammatory burden.

### 4.2. Brodalumab (IL-17RA Antagonist)

A molecular profiling study [3] administered brodalumab 210 mg SC at weeks 0, 1, 2, 4, then biweekly for 12 weeks in 10 Hurley stage II–III patients. Inflammation and pathogenic gene pathway activity decreased. The study lacked clinical endpoints such as HiSCR, used a short follow-up, and was uncontrolled—making translation to clinical efficacy uncertain.

### 4.3. Bimekizumab

The BE HEARD I and II phase 3 RCTs [4] enrolled >1000 patients randomized to multiple bimekizumab dosing regimens or placebo. Both trials met their primary endpoint (HiSCR50 at week 16) with sustained responses to week 48. Strengths include large sample size, randomization, and multiple dosing arms; however, placebo response rates were substantial, and the primary endpoint did not assess durability beyond one year.

### 4.4. Bermekimab (MABp1)

Gottlieb et al. [5] (phase II open-label) and Kanni et al. [9] (open-label extension) assessed bermekimab in anti-TNF failures or ineligible patients. HiSCR rates ranged from 46 to 75% with notable QoL improvements. The absence of a comparator, small sample sizes, and heterogeneity in prior biologic exposure limit generalizability; nonetheless, results support IL-1α inhibition as a promising target.

### 4.5. Anakinra

A small open-label study [10] (*n* = 6) reported improvements in Sartorius scores and global assessments with IL-1 receptor blockade. Lack of a control arm and very small sample size preclude conclusions on comparative efficacy.

### 4.6. Spesolimab

In a phase IIa RCT, Alavi et al. [11] randomized 52 HS patients to spesolimab or placebo. The drug reduced draining tunnels, abscesses, and nodules at week 12. Strengths include randomization and placebo control, though the short trial duration and small cohort limit assessment of long-term benefit and safety.

### 4.7. TNF-α Inhibitors

High-dose, high-frequency intravenous infliximab [7] achieved higher early response rates than historical controls, though findings are limited by non-randomized design and possible selection bias. Mulani et al. [17] found TNF-α inhibition reduced HSS scores in 67 patients, but without a comparator, placebo effects cannot be excluded. Adalimumab, the only FDA-approved biologic for HS, achieves HiSCR in 42–59% [6]. In Kimball et al.’s phase 2 placebo-controlled study, HiSCR was more sensitive than HS-PGA across dosing regimens and Hurley stages, underscoring endpoint choice as a critical trial design consideration.

### 4.8. CJM112

A phase II double-blind RCT [8] found significantly higher HS-PGA response with CJM112 vs. placebo at week 16 (*p* = 0.03). While randomized, the study was exploratory and lacked HiSCR as a primary endpoint, making cross-trial comparisons difficult.

### 4.9. Guselkumab

Kimball et al. [12] tested high-dose guselkumab in 181 patients using multiple regimens. HiSCR rates were numerically higher than placebo but not statistically significant. Pain scores improved significantly. Strengths include multicenter randomization and high-dose exploration; limitations include lack of significance for primary endpoint and potential underpowering for subgroup analyses.

### 4.10. Safety Outcomes

Across studies, safety profiles were acceptable, with upper respiratory infections, injection-site reactions, and gastrointestinal symptoms being the most common. Serious adverse events (AEs) were uncommon. In BE HEARD I/II, serious treatment-emergent AEs occurred in 8% and 5% of patients, respectively. No treatment-related deaths were reported.

## 5. Discussion

This review synthesizes current evidence on targeted pharmacologic agents for HS. Across available studies, TNF-α, IL-17, and IL-1 inhibition demonstrated the most consistent efficacy, though effect sizes, durability, and safety profiles varied between pathways.

TNF-α inhibitors—adalimumab and infliximab—remain the most established biologics in HS. Adalimumab, the only FDA-approved agent, achieves HiSCR in 42–59% of patients in phase 3 trials [6], with durability demonstrated in long-term extension studies. Infliximab, particularly at higher doses or dosing frequency, achieves early clinical responses (up to 70% by week 12) in uncontrolled cohorts [7]. Safety is generally favorable, with infections being the most common adverse event, consistent with the experience in other inflammatory diseases. However, partial response rates and secondary loss of efficacy over time highlight the need for alternative pathways for many patients.

IL-17 inhibitors show robust efficacy in well-powered RCTs. Bimekizumab achieved HiSCR50 in ~48–54% of patients at week 16 in BE HEARD I and II [4], with sustained responses to week 48. Effect sizes were comparable to adalimumab, but data on post-discontinuation relapse and long-term safety remain limited. Secukinumab improved HiSCR and patient-reported outcomes in smaller open-label studies [1,2]; however, standard psoriasis dosing may be insufficient for HS, which has a higher inflammatory burden, indicating that higher or more frequent dosing could be required for optimal response. CJM112 and brodalumab offer mechanistic diversity within the IL-17 pathway but remain in early-phase testing. Safety profiles are acceptable, though IL-17 blockade is associated with candidiasis risk, which warrants monitoring in long-term use.

IL-1 inhibitors appear particularly effective in subsets of patients—especially TNF-α inadequate responders. Bermekimab achieved HiSCR in up to 75% in small open-label studies [5,9], with meaningful improvements in pain and quality of life. Anakinra demonstrated reductions in Sartorius scores and positive patient-reported outcomes [10], though data are limited by very small, uncontrolled cohorts. Safety profiles were generally favorable, with mild injection-site reactions being the most common. The magnitude of response in early studies suggests IL-1 blockade could be a viable second-line biologic strategy once validated in larger trials.

IL-36 inhibition (spesolimab) showed reductions in draining tunnels, abscesses, and nodules versus placebo at week 12 [11], with an acceptable safety profile. The effect size appears modest compared to TNF-α or IL-17 blockade, but its novel target may benefit patients with specific inflammatory phenotypes.

IL-23 inhibition (guselkumab) failed to significantly improve HiSCR despite modest pain reduction [12,13,14,15,16], indicating that elevated cytokine levels do not guarantee therapeutic benefit. The discordance between biomarker rationale and clinical efficacy underscores the importance of integrating translational science with clinical trial design.

Comparative considerations

Across pathways, HiSCR rates in the best-performing regimens typically fall in the 50–70% range in controlled trials, with TNF-α and IL-17 blockade showing the most reproducible results. Durability beyond one year is best established for adalimumab, while long-term data for newer IL-17 and IL-1 agents remain limited. Safety profiles are generally acceptable, but pathway-specific risks (e.g., infections with TNF-α inhibitors, mucocutaneous candidiasis with IL-17 blockade) should inform treatment choice.

Clinical implications and future directions

Given the heterogeneity of HS, a one-size-fits-all approach is unlikely to achieve optimal outcomes. Biomarker-driven stratification—such as cytokine profiling or transcriptomic signatures—may help match patients to the most effective pathway. Trials should prioritize standardized disease activity metrics, including active comparators, and extend follow-up to capture durability and long-term safety. A combination of regimens and sequential therapy algorithms also warrant exploration, particularly in refractory disease. Interdisciplinary research integrating dermatology, immunology, and translational science will be critical for advancing toward truly personalized, mechanistically informed therapy.

## 6. Conclusions

Targeted biologics have transformed the HS therapeutic landscape, with TNF-α, IL-17, and IL-1 inhibitors showing the most consistent efficacy. Other pathways, such as IL-36, remain promising but preliminary, while IL-23 inhibition illustrates the gap between pathogenic plausibility and clinical efficacy. Future drug development should focus on CIDAP-specific mechanisms, incorporate biomarkers for patient selection, and rigorously evaluate durability and safety. Personalized strategies will be essential to achieve durable remission and improve long-term outcomes.

## Figures and Tables

**Table 1:** Summary of targeted biologic therapies for HS by mechanism, trial size, and key efficacy outcomes.

Target/Agent	Key Trials (Design)	*n*	Primary Endpoint	Key Efficacy Findings	Notable Safety Signals
**TNF-α**	Adalimumab (PIONEER I/II, phase 3 RCT) [6]	633	HiSCR wk 12/16	42–59% vs. 26–28% placebo	Infections (mild–moderate)
	Infliximab (open-label) [7]	42	Clinical response wk 4/12	47.6% wk 4; 70.8% wk 12	Infusion reactions, infections
**IL-17A/F**	Bimekizumab (BE HEARD I/II, phase 3 RCT) [4]	1014	HiSCR50 wk 16	48–54% vs. 29–32% placebo; sustained to wk 48	URIs, oral candidiasis
	Secukinumab (open-label) [1,2]	20; 9	HiSCR wk 24	67–70% achieved HiSCR; improved PROs	URIs, mild GI symptoms
	CJM112 (phase 2 RCT) [8]	49	HS-PGA wk 16	↑ responders vs. placebo (*p* = 0.03)	Injection-site reactions
	Brodalumab (molecular profiling) [3]	10	Transcriptomic changes	↓ inflammatory pathways	No major safety events
**IL-1α/IL-1R**	Bermekimab (phase 2 open-label) [5,9]	42; 8	HiSCR wk 12	46–75% achieved HiSCR	Mild infusion reactions
	Anakinra (open-label) [10]	6	Sartorius score	Significant reduction; improved PROs	Injection-site reactions
**IL-36R**	Spesolimab (phase 2a RCT) [11]	52	Lesion count reduction wk 12	↓ draining tunnels, abscesses, nodules vs. placebo	URIs, mild GI symptoms
**IL-23**	Guselkumab (phase 2 RCT) [12,13,14,15,16]	181	HiSCR wk 16	NS difference vs. placebo; pain reduction	URIs, mild GI symptoms

Abbreviations: HiSCR, Hidradenitis Suppurativa Clinical Response; HS-PGA, HS Physician Global Assessment; PROs, patient-reported outcomes; RCT, randomized controlled trial; NS, not significant; URI, upper respiratory infection; GI, gastrointestinal. ↑ indicates increased; ↓ indicates decreased.

## Data Availability

The data that support the findings of this study are available from the corresponding authors upon reasonable request.

## Supplementary Materials

The following supporting information can be downloaded at https://www.mdpi.com/article/10.3390/ijms26188887/s1.
