# Supplementary material for: Targeted Biologic Therapies for Hidradenitis Suppurativa
Source: Int J Mol Sci. 2025 Sep 12;26(18):8887. doi: 10.3390/ijms26188887 (PMC12469770; doi:10.3390/ijms26188887)
Supplement: Supplementary file 1 [file ijms-26-08887-s001.zip › ijms-3762861-supplementary.pdf]

Table S1. Search Strategy.

Database	Full Search Strategy	Entries (total n=607)
PubMed	("Hidradenitis suppurativa"[tiab]) AND ("Targeted therapies" OR "Biologic agents" OR "Monoclonal antibodies" OR "Immunotherapy" OR "Molecular targeted therapy" OR "Precision medicine" OR "TNFα" OR "IL-17" OR "IL-23" OR "IL-1β" OR "IL-1α" OR "anti-androgen" OR "IGF-1" OR "PPAR inhibitor")	n=427
Embase	('Hidradenitis suppurativa') AND ('Targeted therapies' OR 'Biologic agents' OR 'Monoclonal antibodies' OR 'Immunotherapy' OR 'Molecular targeted therapy' OR 'Precision medicine')	n=174
ClinicalTrials.gov	Condition/disease: Hidradenitis Suppurativa; Other terms: Apocrine; Intervention/treatment: Targeted Therapy	n=6
